# EMA perspective on Diagnosis of Alzheimer's

**DOI:** 10.1002/alz70857_107832

**Published:** 2025-12-26

**Authors:** Steffen Thirstrup

**Affiliations:** ^1^ European Medicines Agency, Amsterdam, Amsterdam, Netherlands

## Abstract

**Background:**

Regulatory agencies approval of medicines is always associated with a guidance to the prescriber and patient on the correct use. This include among other a detailed description of the target indication, ie the population for which the product has been shown to have an acceptable benefit:risk ratio. The population of the approved target indication is reflected in the patient population in which the supporting clinical evidence has been generated, which then again is linked to the design and conduct of therapeutic confirmatory clinical trials.

**Method:**

Review of current EMA policy

**Results:**

Having globally agreed diagnostic criteria for a certain condition/disease is favouring global drug development and global regulatory alignment. Without such alignment clinical trials many have to be replicated or designed to meet different diagnostic criteria to meet regulatory expectations.

**Conclusion:**

This intervention will briefly outline the position of the European Medicines Agency on the above challenges with special attention to the current debate on diagnostic criteria for Alzheimer's Disease

